# A quantum sensing metrology for magnetic memories

**DOI:** 10.1038/s44306-024-00016-5

**Published:** 2024-06-03

**Authors:** Vicent J. Borràs, Robert Carpenter, Liza Žaper, Siddharth Rao, Sebastien Couet, Mathieu Munsch, Patrick Maletinsky, Peter Rickhaus

**Affiliations:** 1 Qnami AG, Muttenz, Switzerland; 2 Imec, Kapeldreef 75, 3001 Leuven, Belgium; 3 Department of Physics, University of Basel, Basel, Switzerland

**Keywords:** Quantum metrology, Electronic and spintronic devices

## Abstract

Magnetic random access memory (MRAM) is a leading emergent memory technology that is poised to replace current non-volatile memory technologies such as eFlash. However, controlling and improving distributions of device properties becomes a key enabler of new applications at this stage of technology development. Here, we introduce a non-contact metrology technique deploying scanning NV magnetometry (SNVM) to investigate MRAM performance at the individual bit level. We demonstrate magnetic reversal characterization in individual, <60 nm-sized bits, to extract key magnetic properties, thermal stability, and switching statistics, and thereby gauge bit-to-bit uniformity. We showcase the performance of our method by benchmarking two distinct bit etching processes immediately after pattern formation. In contrast to ensemble averaging methods such as perpendicular magneto-optical Kerr effect, we show that it is possible to identify out of distribution (tail-bits) bits that seem associated to the edges of the array, enabling failure analysis of tail bits. Our findings highlight the potential of nanoscale quantum sensing of MRAM devices for early-stage screening in the processing line, paving the way for future incorporation of this nanoscale characterization tool in the semiconductor industry.

## Introduction

The ability to store information magnetically has been a major enabler of the digital revolution^1^. To improve storage density, and energy efficiency, magnetic bits have become smaller and denser^2^, presenting an ever-increasing challenge for metrology tools—one that is intensifying with the push towards exotic materials, such as 2-D and antiferromagnetic magnets, which have small surface moments^3,4^. Emerging, ultrasensitive, nanoscale magnetic quantum sensors^5^ offer a unique opportunity to address this challenge due to their highly competitive sensing characteristics. To demonstrate the advantage of such quantum metrology in an industrially relevant context, and scale, spin transfer torque–magnetic random access memory (STTMRAM) is an ideal candidate. STT-MRAM is one of the most promising next-generation, non-volatile memory architectures and one that is already in production for embedded flash replacement at N2X nodes^6,7^. It is constructed around a magnetic tunnel junction (MTJ), consisting of two magnetic layers, the free layer (FL) and the reference layer (RL), and a tunnel barrier. The role of the FL is to act as the storage layer, while the RL generates a spin-polarized current. It is this spin-polarized current that, via spin transfer torque (STT), can switch the FL and read the bit state via the tunneling magnetoresistance ratio (TMR) effect^8,9^.

Due to the interfacial nature of both the TMR and STT effects, uniformity is a significant challenge for STT-MRAM. This is further exacerbated by the nanoscale dimensions of the magnetic layers, with typical target layer thicknesses, and bit sizes, of <2 and <60 nm, respectively. In particular, the data retention, related to the energy barrier required to change the magnetic orientation of the FL, is defined by the aniostropy and volume of the layer. Therefore, the energy to erase, or write, the FL is volumetric and thus scales in quadrature with the diameter^10^. This makes device variability especially sensitive to variations in the etch conditions.

While STT-MRAM is now in high volume manufacturing at several foundries, expanding the application space is also linked to controlling the distributions of device properties. This requires having individual access to out-of-distribution bits for failure analysis. Therefore, metrology tools to measure all these parameters are required in both in-line production and process development. Magneto-optical Kerr effect (MOKE) magnetometry^11–15^ and current in-plane tunneling (CIPT)^16^ are the most common methods. CIPT is capable of measuring the as-deposited film properties. Individual bits characterization is only possible at the end of the integration process, meaning a long feedback loop from process to performance. In addition, magnetization of individual bits can’t be measured with the current techniques. Scanning nitrogen vacancy magnetometery (SNVM) can fill this key metrology gap (see Table 1), due to its high spatial resolution and magnetic field sensitivity the magnetic properties of each bit can be characterized, providing rich information that could improve the understanding and therefore the development of STT-MRAM. In addition, by measuring the state of each bit, switching distributions and thermal stabilities can also be obtained without the need to connect the device electrically. A standard STT-MRAM process flow consists of at least two additional steps following MTJ pillar patterning, namely, passivation and metallization. The metallization module creates a top contact to the patterned MTJ to enable electrical characterization, which is typically fabricated by the dual damascene process. Depending on the integration scheme involved, the number of metal layers in the top contact can range from as few as 2 copper metal layers up to 5 copper metal layers. As each layer grown by the dual damascene process involves 2 masks for lithography and patterning, the total number of masking operations ranges from 4 masks (for 2 metal layers) up to 10 additional masks (for 5 metal layers). As a result, significant costs are incurred in enabling electrical characterization of the patterned MTJ devices at end-of-line. In comparison, enabling a methodology to evaluate device properties immediately after MTJ patterning can save in both development time and costs. Furthermore, the array-level retention assessment at end-of-line is typically done by thermal bake experiments that can go up to 2 weeks in measurement time^17^. In contrast, the SNVM technique takes ~1.5 h to perform a magnetic field scan of a 2 kB array and extract the array-level retention, thereby significantly reducing the test time required.

**Table 1:** Comparison of SNVM to other metrology techniques used to characterize STT-MRAM

	Acquisition speed	Fabrication stage	Metallization modules required?	Resolution	Type of information
CIPT	~30 s/point	Before patterning	Yes	~60 μm	Film resistances
MOKE	<20 s^a^	Before/after patterning	No	>~40–60 μm	Magnetic properties
SNVM	~3 s/bit^b^	After patterning	No	~20–100 nm	Magnetic properties, switching distributions
Electrical characterization	~1 s/bit	End of line	Yes	Individual bits	Resistances, switching distributions

Acquisition speed and type of information can vary for every technique depending on the measurement protocol; the indicated values correspond to the most common protocols.

^a^This value correspond to a complete hysteresis loop. A single MOKE data point can be obtained in few ms.

^b^SNVM acquisition time could be increased up to AFM speed. Ultimately, a single magnetic field measurement (~ms) should be enough to identify the bit state. See more details in “Conclusion.”

Alternative approaches to measuring single pillars are conductive atomic force microscopy (cAFM) and magnetic force microscopy (MFM)^18^. While cAFM enables electrical measurements after patterning, direct contact with a pillar is required which precludes straightforward use for in-line metrology. Conversely, MFM can be used to map arrays, similar to the approach in this work. However, the resolution of MFM is limited by the tip diameter which is typically >30 nm and the magnetic field sensitivity is typically lower. In addition, the technique is invasive and tip-to-tip variations can cause non-reproducible results, further limiting its application as an in-line measurement technique.

In this work, we use SNVM to estimate the retention, and characterize the bit-to-bit uniformity of two etch processes^19^. We describe and apply a statistical method that is well suited to analyze the switching uniformity based on SNVM data. We find that while both processes result in significant bit-to-bit variations, the uniformity is improved with the optimized etch process. A single NV stray field map is sufficient to measure the improved uniformity. Importantly, our results are consistent with prior electrical characterization studies of the same processes^19^, which confirms the validity of our conclusion and the usefulness of SNVM as a future in-line characterization tool.

## Results

**Fig. 1:**
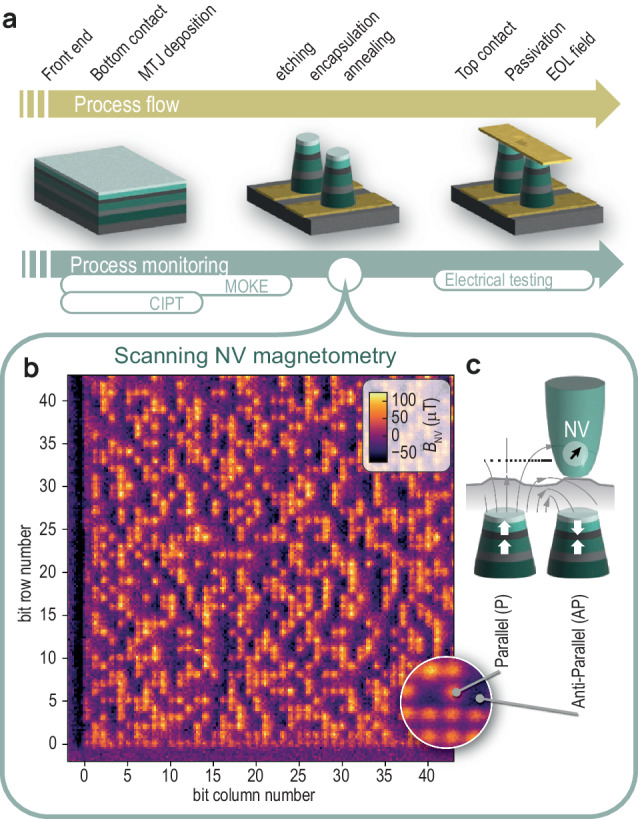
SNVM as metrology tool for STT-MRAM. **a** Process flow of STT-MRAM fabrication, including process monitoring. **b** SNVM map of 45 × 45 bits (10 × 10 μm) after encapsulation. Bits in the anti-parallel (AP) state appear dark, bits in the parallel (P) state appear bright. **c** P and AP bit configurations generate distinct stray field patterns (gray lines). The NV probe measures their projection onto the NV quantization axis (black arrow) at the flying distance of the NV probe.

An example of an SNVM measurement on an STT-MRAM array is shown in Fig. 1b, with details given in the “Methods” section. The image contains 45 × 45 bits and demonstrates the ability of SNVM to distinguish the logical state of individual bits. The scan took approximately 1.5 h. The image is obtained by continuously scanning the sample under an NV probe recording the projection of the local magnetic stray field onto the NV quantization axis, see Fig. 1c: bits in the parallel (P) and antiparallel (AP) states (c.f. Fig. 1c, right) yield comparatively high and low stray field magnitudes, leading to a bright and dark imaging contrast, respectively.

**Fig. 2:**
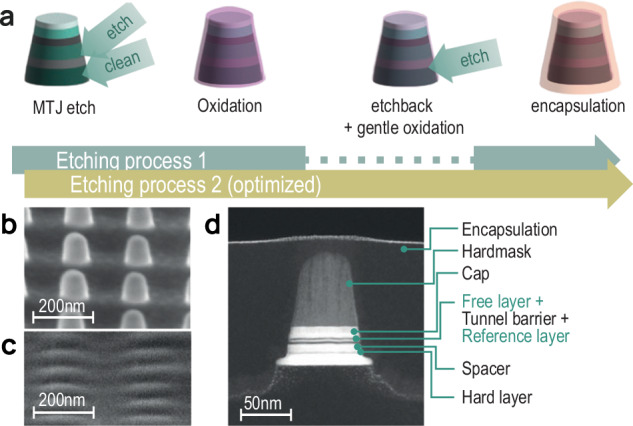
STT-MRAM. **a** The quality of etching Processes 1 and 2 are compared in this work. Etching Process 2 features an additional etchback and gentle oxidation step after the first oxidation. **b** Scanning electron microscope images of the MRAM pillars before and **c** after encapsulation. **d** Transmission electron microscope image of an MRAM pillar.

In order to demonstrate the key impact SNVM characterization can have on STT-MRAM development, two wafers were fabricated in an industrial equivalent, 300 mm wafer process. As one of the most critical steps in controlling STT-MRAM yield is the etch process, characterization of this step was chosen as the main focus of this study. Thus two wafers were prepared using different etch processes, see Fig. 2a. Process 1 consists of MTJ etching, oxidation, and encapsulation. In Process 2, an additional etchback and gentle oxidation step is introduced^19^. Previous work has shown in electrically connected devices that Process 2 increases yield and decreases the bit-to-bit performance variation^19^.

The pillars were designed with a nominal 60 nm diameter and a pitch of 200 nm, but after patterning, the physical diameter of the pillars is typically reduced to ~45 nm. These dimensions are fully aligned with current and near-future application domains of STT-MRAM technology. The MTJ tri-layer consists of a CoFeB FL, an MgO tunnel barrier and a CoFeB RL. SEM images before and after encapsulation are shown in Fig. 2b, c, alongside a typical TEM image (Fig. 2d) where the key layers are labeled. The samples were encapsulated with a SiN layer to avoid further exposure of the pillar to oxygen.

**Fig. 3:**
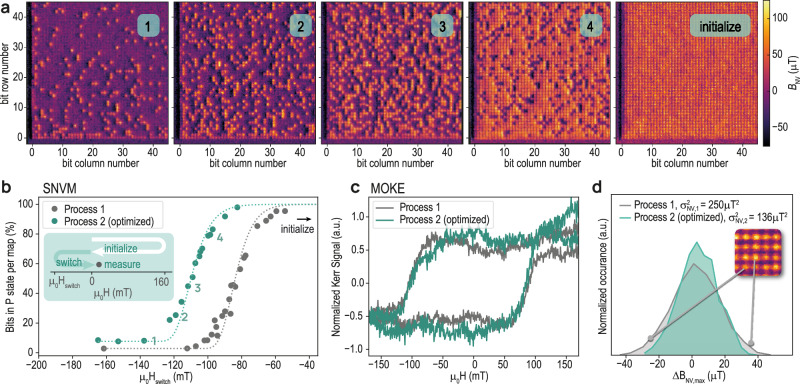
Quantitative determination of Δ and *H*_*k*_. **a** SNVM maps, obtained for different switching fields *μ*_0_*H*_switch_. **b** For each map in (**a**), the percentage of P bits is plotted versus *μ*_0_*H*_switch_, which is fitted to obtain *H*_*k*_ and Δ, inset: measurement routine where the bits are first initialized at *μ*_0_*H* = 160 mT followed by a switching field *μ*_0_*H*_switch_. The measurements are performed at *μ*_0_*H* = 2 mT. **c** Polar MOKE hysteresis loops of the FL measured on the same wafers. **d** Histogram of the maximum measured stray field per bit in P state, shifted with respect to the median of the distribution, $\Delta {B}_{{{{\rm{NV}}}},\max }$.

We prepare and manipulate the bit states of the MRAM pillars by exposing them to an out-of-plane bias magnetic field, *μ*_0_*H*, of varying strength. The resulting SNVM images in Fig. 3a are obtained for increasing values of *μ*_0_*H*, using the measurement protocol illustrated in the inset to Fig. 3b: The bits are initialized in the P state by sweeping *μ*_0_*H* to 160 mT. We then switch a percentage of the bits to the AP state by sweeping to a negative field, *μ*_0_*H*_switch_, and then perform SNVM characterization in a small bias field *μ*_0_*H* = 2 mT. Images in panels 1–4 in Fig. 3a are obtained with increasing values of *μ*_0_*H*_switch_ where we observe a decreasing number of bits in the AP state. The image in panel 5 was measured directly after the initialization step, where all bits are aligned in the P state. Interestingly, we can initialize the array in a complete P state, but not in a complete AP state (Fig. 3a, panel 1), even when applying fields well above the average coercive field of the bits. This is seen in Fig. 3c where the preferred orientation is the P state. This asymmetry can be explained by the non-compensated stray field generated by the RL and felt by the FL. The asymmetry is particularly pronounced for bits at the edge of the bit array (row 1 and column 1, as well as row 2 and column 2), which indicates undesired magnetic properties of the bits close to the edge. Such difference may be explained by altered etching conditions at the edges of the arrays. Alternatively, the local stray fields may affect the neighboring pillars. However, given the significant spacing of the pillars (the edge-to-edge spacing >3× the pillar diameter), such a cross-talk is unexpected^20,21^. This observation underlines the importance of ultrasensitive local metrology of MRAM devices as conclusive measurements on the edge switching statistics remain, to our knowledge, elusive. In the following, we will exclude rows 1–4 and columns 1–4 from our data analysis, due to their unusual behavior.

To further analyze and quantify the bit switching process, we plot the percentage of parallel bits as a function of *μ*_0_*H*_switch_ in Fig. 3b. The field at which 50% of devices switch is found to be ~85 and ~110 mT for etch Processes 1 and 2, respectively. This is in excellent agreement with the coercive field, *μ*_0_*H*_*c*_ of the film (defined as half width of the MOKE hysteresis loop), for which we find *μ*_0_*H*_*c*_ ~ 110 mT by polar MOKE magnetometry (Fig. 3c). These results provide another illustration of the advantage SNVM over traditional approaches, since SNVM clearly distinguishes the performance of the two etch processes, while MOKE does not. The latter being limited by the large laser spot size employed, that leads to averaging over ~10^6^ bits and includes peripheral magnetic material that is in the field of the measurement. Therefore, while the polar MOKE is well suited to measure the overall magnetic properties of thin films or bit ensembles, it is not sensitive to single devices or details of the performance distribution. Note that while micron-range spot sizes are available in MOKE magnetometers, this is not sufficient to resolve individual bits and to the author’s knowledge has never been applied to industrially relevant MRAM devices.

We proceed by fitting the SNVM data (dashed line, Fig. 3b) using the domain wall mediated reversal (DWMR) model^22,23^ in order to calculate the retention value (Δ)

$$\Delta := \frac{{E}_{b}}{{k}_{{\rm{B}}}T},$$

where *E*_b_ is the energy barrier to switch between the P and AP states, *k*_B_ is the Boltzmann constant, and *T* is the temperature. Fitting our SNVM data, we obtain values Δ = 49 and Δ = 54 for etch Processes 1 and 2, respectively, indicating that the optimized process has a higher thermal-stability. In addition to the retention, the anisotropy fields (*H*_*k*_) are calculated during the fitting and are found to be *μ*_0_*H*_*k*_ = 440 ± 2 mT for Process 1 and *μ*_0_*H*_*k*_ = 533 ± 2 mT for Process 2. For further details on the fitting, see “Methods.”

Due to the single-bit sensitivity of SNVM, we can furthermore extract valuable information by assessing the uniformity of the measured stray magnetic field. Figure 3d shows the distributions of the maximum measured stray field ${B}_{{{{\rm{NV}}}},\max }$ per bit in the P state for the two etch processes. It is clearly seen that Process 2 has a narrower distribution with a stray-field variance ${\sigma }_{{{{B}}}_{{{{\rm{NV}}}}}}^{2}=136\,\upmu$T^2^, as compared to that of Process 1, where ${\sigma }_{{{{B}}}_{{{{\rm{NV}}}}}}^{2}=250\,\upmu$T^2^. This indicates that Process 2 has a higher bit-to-bit uniformity, in agreement with earlier findings^24^.

**Fig. 4:**
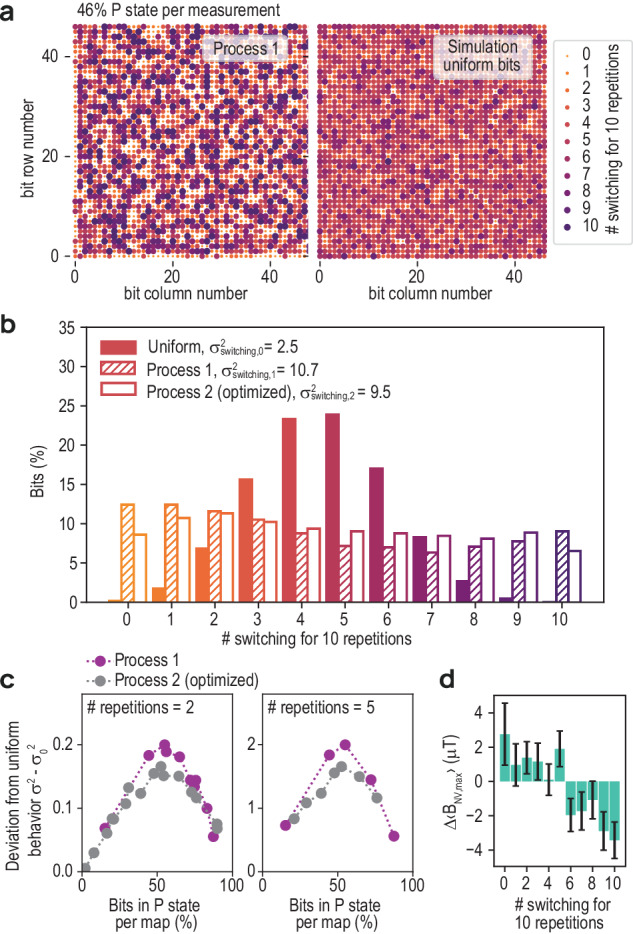
Array uniformity. **a** The map on the left shows, by dot size and color, how often a bit has switched during 10 repetitive measurements. For each repetition, a map, as shown in Fig. 3a with 46% of bits in P state, is taken. The simulation illustrates how such a map would appear for perfectly uniform bits. **b** Density histogram of the maps in (**a**). The results are compared to a binomial distribution (purple) reflecting the expected outcome for perfectly uniform bits. **c** Deviation from uniform behavior (${\sigma }_{{{{\rm{switching}}}},i}^{2}-{\sigma }_{{{{\rm{switching}}}},0}^{2}$) as a function of percentage of bits in P states for each individual measurement, we chose to show 2 and 5 repetitions. **d** Stray field in fully initialized map of Process 2, Δ*B*_NV,max_ (see in Fig. 3d), as a function of # switching of the corresponding bits. The stray field is averaged over all bits that show the same number of switching events. Error bars indicate twice the standard error.

In addition, we propose and demonstrate an alternative method to evaluate the homogeneity of the bit switching process across the array, which is especially well suited to analyze SNVM data and any other dataset that deals with a large array of bits and a limited amount of switching repetitions. The method will confirm that ${\sigma }_{{{{{\rm{B}}}}}_{{{{\rm{NV}}}}}}^{2}$ is indeed a valid qualifier for bit-to-bit performance uniformity. For this, we evaluate how often each bit successfully switches its state when exposing it several times to the same initialization and switching conditions. We repeat initialization, switching and mapping 10 times by applying a value of *μ*_0_*H*_switch_ for which almost half of the bits (46%) remain in the P state for a single switching cycle. The statistics of switching events for a full bit-array is shown in Fig. 4a (left) for etch Process 1, where the number of successful switches is color-coded in the dots representing the bits. In comparison to a simulation of bits obeying a binomial distribution (Fig. 4a, right), we note that a significant fraction of bits on the sample switch never (small yellow dots) or every time (large purple dots). This non-binomial behavior is particularly pronounced at the edge of the array, as discussed earlier.

For a quantitative analysis, we show the corresponding density histogram in Fig. 4b. For Process 1, we observe that 12% (9%) of the bits switch zero (ten) times. Process 2 has a narrower distribution with 8% (6%), of zero (10) switching events. For comparison, in an array following a binomial distribution, only 0.2% (0.04%) of bits would switch zero (10) times. To quantify the non-uniformity, we extracted the variance of the distributions, and obtain ${\sigma }_{{{{\rm{switching}}}},0}^{2}=2.5$ for the binomial distribution and ${\sigma }_{{{{\rm{switching}}}},1}^{2}=10.7$ and ${\sigma }_{{{{\rm{switching}}}},2}^{2}=9.3$ for Processes 1 and 2, respectively. Although the deviation from the binomial distribution is large, there is clearly a more uniform behavior of the optimized Process 2 as compared to Process 1.

To confirm this observation, we repeat this experiment by changing *μ*_0_*H*_switch_ to obtain different overall switching probabilities. In Fig. 4c, we show the deviation from the uniform behavior, ${\sigma }_{{{{\rm{switching}}}},i}^{2}-{\sigma }_{{{{\rm{switching}}}},0}^{2}$ (with *i* ∈ 1, 2), as a function of the average percentage of bits in the P states for 2 and 5 repetitions. The graphs show a consistently high deviation, which peaks at a switching probability ~50%, which therefore marks the condition where the difference between the two processes becomes most evident. We note that, Process 1 consistently shows a stronger deviation from binomial behavior than the optimized Process 2, as is already apparent after two repetitions. The deviation from binomial switching is due to significant bit-to-bit variations of the relevant magnetic properties (magnetization, volume, shape, anisotropy) which is less pronounced for the optimized Process 2.

Importantly, the conclusion of these switching statistics agree well with the pattern observed in the stray field distribution (see Fig. 3d). For Process 2, the comparison of the average stray field measured from each bit to its switching behavior (Fig. 4d), shows that those bits that are more (less) likely to switch produce, on average, a higher (smaller) magnetic stray field. This indicates that stray field distributions obtained with sensitive magnetic field measurements on initialized arrays can be used to retrieve information on the uniformity of the switching behavior.

**Fig. 5:**
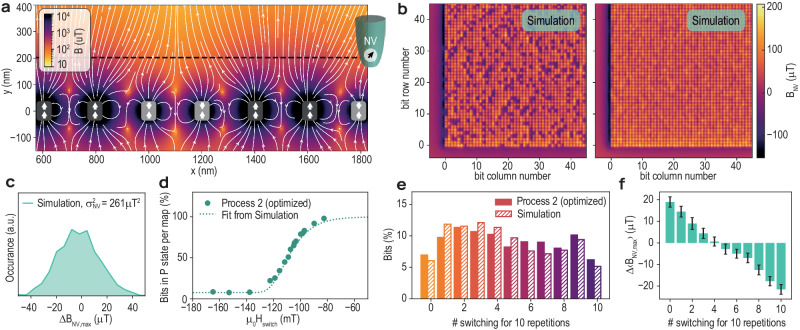
Magnetization distribution. **a** Schema of the magnetic field obtained from a simulation for 7 pillars in different states (P or AP). The dashed horizontal line corresponds to the NV flying distance. **b** NV maps obtained by simulations for 80% and 100% of the bits in the P state. **c** Magnetic field distributions of maps in (**b**). **d** Percentage of P bits versus *μ*_0_*H*_switch_ for Process 2 (dots) and for the simulation (dotted line) **e** Density histogram obtained after repeating the measurement 10 times at 50% of switching probability. **f** Stray field in fully initialized simulated maps, Δ*B*_NV,*max*_, as a function of # switching of the corresponding bits. The stray field is averaged over all bits that show the same number of switching events. Errorbars indicate twice the standard error.

Finally, in order to understand the variations of the stray fields in terms of pillar properties, such as magnetization and critical dimension, we present simulations using the Magpylib library^25^. Here, the devices are modeled as cylindrical layers of different thicknesses and saturation magnetizations (see Fig. 5a). The values for this simulation were taken from measurements made on non-patterned samples (data not shown, for further details, see “Methods”). The magnetic stray field is then calculated for a large array of pillars and projected onto the NV quantization axis at a height corresponding to the NV flying distance. This results in simulated maps that compare well to the SNVM measurements. Figure 5b shows an example of a simulation in which we considered a normal distribution of the magnetization in the FL with a standard deviation of 0.235 MA/m, a normal distribution in the critical dimension with 0.8 nm of standard deviation and normal distributions in the magnetization directions with standard deviations of 10° and 5° for the RL and FL, respectively. These values are taken according to recent work measured on similar samples to investigate the dispersion of easy-axes orientations^26^. Taking these into account, a stray field histogram is generated in Fig. 5c, which we find to be in good agreement with the one obtained from SNVM data. Furthermore, assuming these distributions, the switching probability and the density histogram measured for etch Process 2 are well reproduced (Fig. 5d, e, respectively). Finally, in Fig. 5f, we plot the mean magnetic stray field as a function of the number of switching events, in order to reproduce Fig. 4d. Despite a difference in absolute value between experiment and simulation, the same trend is observed. Our simulation suggests that the non-binomial behavior is related to the magnetic and structural variations among the pillars, such as the saturation magnetization, the magnetization angle, and the pillar diameter. These variations are directly related to the key metrics for MRAM device health, such as the thermal stability (see “Methods”) and read/write currents. This being measurable at the stage of the pillar etch is a significant step towards a rapid, early, detection of MRAM process failures.

## Discussion

In conclusion, we demonstrate that SNVM is a powerful technique for the measurement and characterization of MRAM device properties early in the manufacturing process, directly after the devices etching step. Furthermore, high-resolution imaging of MRAM pillars of industrially relevant diameters is achieved, even with encapsulation layers present. Our data are obtained with single-pillar resolution, a capability which is not available to present-day in/off-line metrology techniques. The established methodology can be used for both process failure analysis early in the device fabrication (post-etch), enabling accelerated process development, in particular for new design where bit environment might change and localized magnetization map provide key failure mode analysis. With increased measurements speed, the method could also be used as process control, enabling early detection of out of control stack or etch process for instance. The SNVM scan speed could be improved few orders of magnitude in future measurements by reducing the number of pixels per bit (even 1 pixel/bit), number of ODMR frequencies to measure (even 1 frequency), the integration time and improving the hardware–software communication speed. With these improvements the SNVM scanning speed should be comparable to AFM speed. The quality of our SNVM images enables a quantitative analysis of the bit uniformity through a single stray-field map or a measurement of the switching behavior. The latter can then be used as a method to extract the thermal stability with a sensitivity at the single-bit level. Finally, the flexibility of this technique is such that measurements normally limited to chip level could be enabled at an early stage. For example, by incorporating localized heating or perturbation fields, the probability of data loss in different application environments can be explored. These combined assets show the strength of nanoscale quantum metrology for addressing the magnetic ordering of patterned, magnetic, systems that are now in industry-scale production. Furthermore, the ability to sense even smaller magnetic fields such as those emerging from unsaturated spins in antiferromagnets^27,28^ puts quantum sensing and the hereby demonstrated methodology in a unique position for meteorology on a wide range of future magnetic memory architectures.

## Methods

### Fabrication method

The deposition of the MTJ films was carried out by magnetron sputtering (Canon-Avelva EC7800) on 300 mm, thermally oxidized, Si wafers. The thicknesses of all the layers were controlled through calibration of the deposition rates which, depending on the deposition conditions, are in the range of 0.1–1 Å/s. To simulate back end of line thermal budget conditions, the films were post-annealed at 400 °C in a vacuum environment for 30 min (TEL-MS2 MRT5000). These films were then patterned into large arrays of pillars with a design (final) diameter of 60 nm (45 nm), with a center-to-center separation of 200 nm. The arrays were printed using immersion lithography, followed by patterning via ion beam etching. The different etching approaches involved careful control of the side-wall etching and oxidation. For a comparison of the two processes, please see previous work published by the imec team^19^.

### Scanning NV magnetometry

Our SNVM measurements were carried out on dies diced from the 300 mm wafer using a diamond saw with an accuracy of 45 μm. SNVM maps were recorded using the Qnami ProteusQ microscope. The SNVM technique is based on a nitrogen vacancy (NV), which is implanted very close to the apex of a diamond pillar^29^. The NV center is highly sensitive to external magnetic fields^30^. While scanning the SNVM pillar over an MRAM array, the local magnetic stray field pattern is recorded. This stray field is taken at a distance of ≈150 nm to the FL. The distance includes the cap, the hardmask an encapsulation and additionally the flying distance of the NV center on top of the surface (≈50 nm). The measured signal corresponds to the stray field vector at this distance, projected onto the NV-axis, which is tilted by *ϕ* = 54. 5° with respect to the *z*-axis and has an angle in the *x*–*y* plane of *θ* ≈ 0° with respect to the *x*-axis. This projected magnetic field leads to a Zeeman splitting of the NV $\left\vert {m}_{s}\pm 1\right\rangle$ state. We follow the position of the $\left\vert {m}_{s}=-1\right\rangle$ state by recording the NV fluorescence versus applied microwave frequency in the 2.8 GHz range (optically detected magnetic resonance, ODMR^29^). Each of the scans contains 100 × 100 pixels and took approximately 1.5 h.

### Data analysis and simulations

To obtain the bits array from the magnetic field map (e.g., Fig. 1b) we prepared a script that finds the optimized grid in which the pillars are centered inside the grid squares. Then, depending on the mean value of the magnetic field each bit is classified as either 0 or 1.

To fit the data of Fig. 3b and obtain the Δ values we used the well-known Néel–Brown relaxation model

1 $$P({H}_{switch})=1-exp\left[\frac{-{f}_{0}{H}_{switch}}{R}exp\left[-\Delta ({H}_{switch})\right]\right],$$

where the *f*_0_ is the attempt frequency (1 GHz), *R* is the field sweep rate (5 mT/s) and Δ(*H*_switch_) is calculated assuming the domain wall mediated reversal (DWMR) model^22,23^ as

2 $$\Delta ({H}_{{\rm{switch}}})=\frac{1}{{k}_{{\rm{B}}}T}\left[{\sigma }_{{\rm{dw}}}{l}_{{\rm{dw}}}\tau -{\mu }_{0}{H}_{{\rm{switch}}}{M}_{s}\tau \left[{A}_{+{\delta }_{{\rm{w}}}}+{A}_{-{\delta }_{{\rm{w}}}}\right]\right],$$

where *k*_B_ is the Boltzmann constant, *T* is the temperature (298 K), *σ*_dw_ is the domain wall energy density, *l*_dw_ is the domain wall length, *τ* and *M*_*s*_ are the thickness (1.2 nm) and the magnetization (1.178 MA/m) of the FL, respectively, and ${A}_{-{\delta }_{{\rm{w}}}/2}$ and ${A}_{+{\delta }_{{\rm{w}}}/2}$ are the reversed and unreversed areas excluding the domain wall width *δ*_W_, which can be estimated with the following relation^23^:

3 $${\delta }_{{\rm{W}}}=2\log 2\sqrt{2{A}_{{\rm{ex}}}/({M}_{s}{H}_{k})}.$$

Finally, the thermal stability is directly obtained as

4 $$\Delta =\frac{{\sigma }_{{\rm{dw}}}D\tau }{{k}_{{\rm{B}}}T}=\frac{\sqrt{8{M}_{s}{H}_{k}{A}_{{\rm{ex}}}}D\tau }{{k}_{{\rm{B}}}T},$$

where *H*_*k*_ is the magnetic anisotropy, *A*_ex_ is the exchange stiffness (4.5 pJ/m), and *D* is the critical dimension of the pillar (38.9 nm). All the variables but the magnetic anisotropy *H*_*k*_ are assumed based on those measured for our samples, for detailed measurements please see ref. ^15^. To account for the impossibility to switch all the bits at large negative magnetic fields, we also divide Eq. (1) by a normalization constant. Under the above considerations, the anisotropy fields (*H*_*k*_) obtained by fitting are *μ*_0_*H*_*k*_ = 440 ± 2 mT for Process 1 and *μ*_0_*H*_*k*_ = 533 ± 2 mT for Process 2, which correspond to thermal stabilities (Δ) of 49 and 54, respectively. The domain wall widths (*δ*_W_) are also estimated to be 6 and 5 nm for Processes 1 and 2, respectively, a bit smaller than the estimated by Mihajlovic et al.^23^ for a slightly different film stack (~12 nm).

To perform the simulations we used the Magpylib library^25^. In these simulations, the consecutive pillars are separated by 200 nm and each pillar is described with three cylinders with different thicknesses, diameters and magnetizations representing the FL, RL, and HL. For the simulation shown in Fig. 5, we considered a thickness of 1.2, 1.4, and 3.8 nm and a magnetization of ±1.175, +0.790, and +0.550 MA/m for the FL (P or AP state), RL, and HL, respectively. For the critical dimension of the different pillars, we assumed a normal distribution centered at 38.1 nm with a standard deviation of 0.8 nm, which we base on SEM images similar to the one shown in Fig. 2b. For FL and RL, we also considered a normal distribution in the magnetization direction centered at 0° (out of plane) with a standard deviation of 10° and 5°, respectively. For the FL, a normal distribution in the absolute value of the magnetization centered at 1.175 MA/m with a standard deviation of 0.235 MA/m was also considered. Finally, to mimic the NV center, the magnetic field was measured at 151 nm from the FL and at 54. 5° with respect to out-of-plane direction.

## Data Availability

The data used in this work are available from the corresponding author upon reasonable request.
